# ~3-nm ZnO Nanoislands Deposition and Application in Charge Trapping Memory Grown by Single ALD Step

**DOI:** 10.1038/srep38712

**Published:** 2016-12-19

**Authors:** Nazek El-Atab, Farsad Chowdhury, Turkan Gamze Ulusoy, Amir Ghobadi, Amin Nazirzadeh, Ali K. Okyay, Ammar Nayfeh

**Affiliations:** 1 Institute Center for Microsystems – iMicro, EECS, Masdar Institute of Science and Technology Abu Dhabi, United Arab Emirates; 2 Institute of Materials Science and Nanotechnology, Bilkent University, 06800 Ankara, Turkey; 3 UNAM-National Nanotechnology Research Center, Bilkent University, 06800 Ankara, Turkey; 4 Department of Electrical and Electronics Engineering, Bilkent University, 06800 Ankara, Turkey

## Abstract

Low-dimensional semiconductor nanostructures are of great interest in high performance electronic and photonic devices. ZnO is considered to be a multifunctional material due to its unique properties with potential in various applications. In this work, 3-nm ZnO nanoislands are deposited by Atomic Layer Deposition (ALD) and the electronic properties are characterized by UV-Vis-NIR Spectrophotometer and X-ray Photoelectron Spectroscopy. The results show that the nanostructures show quantum confinement effects in 1D. Moreover, Metal-Oxide-Semiconductor Capacitor (MOSCAP) charge trapping memory devices with ZnO nanoislands charge storage layer are fabricated by a single ALD step and their performances are analyzed. The devices showed a large memory window at low operating voltages with excellent retention and endurance characteristics due to the additional oxygen vacancies in the nanoislands and the deep barrier for the trapped holes due to the reduction in ZnO electron affinity. The results show that the ZnO nanoislands are promising in future low power memory applications.

Nanoislands grown on solid surfaces constitute a nanostructured surface system with tunable electronic properties and various applications in high performance electronic, optoelectronic and photonic devices. In photovoltaics, nanoislands are shown to act as a light trapping scheme to enhance the solar cell’s efficiency^1^,^2^. A multimode resistive switching is also demonstrated in single nanoisland systems^3^. Nanoislands can be deposited by different techniques including chemical vapor deposition^4^, sputtering^5^, in addition to predefining the nucleation sites using nanoindentation^6^, etc. Moreover, Zinc-Oxide (ZnO) has recently received considerable attention by industry and research due to its excellent chemical and physical properties such as large bandgap, high exciton binding energy (60 meV), high transparency, high electron mobility, and high mechanical, chemical, and thermal stability at room temperature. These unique properties turn ZnO into a very attractive material with potential in numerous applications in electronics, optoelectronics, photocatalysis and laser devices^7^,^8^,^9^,^10^,^11^,^12^,^13^. ZnO is most commonly deposited by either Atomic Layer Deposition (ALD) or physical vapor deposition such as sputtering. Within the past few years, ALD has gained world-wide attention for manufacturing conformal layers with thickness in the nanometer range, particularly for microelectronic applications^14^,^15^,^16^. However, very recently, it has been shown that the growth per cycle in ALD for the first 20 cycles can be less than a monolayer per cycle which promotes the growth of nanoislands described by a Volmer-Weber growth mechanism^17^. After multiple ALD cycles, the islands start to coalesce and form a continuous film. However, in the ultra-thin-film regime, the ALD films are rough and not conformal to the initial substrate^17^. In the present study, ~3-nm ZnO nanoislands deposited by ALD are demonstrated. Their physical and electronic properties are analyzed and their effect on the performance of Metal-Oxide-Semiconductor Capacitor (MOSCAP) charge trapping memory devices fabricated by single ALD step is investigated. In our previous works, we have demonstrated memory devices with different charge trapping layers such as graphene nanoplatelets^18^, InN nanoparticles^19^, ZnO nanoparticles^20^, Si-nanoparticles^21^,^22^,^23^, etc. However, all of these nanomaterials have been deposited by spin/dip coating or drop-casting and therefore the memory was not grown by a single ALD step which would greatly reduce the possibility of contamination during the fabrication process. Moreover, we have previously shown memory devices with a continuous ZnO layer deposited by ALD with 2-nm^24^ and 4-nm^19^ thicknesses. The achieved memory window was negligible even at very large program/erase voltages (10/−10 V) which show that the continuous ZnO layer deposited by ALD shows small charge trap states density unlike ALD deposited ZnO nanoislands.

## Results

### ZnO Nanoislands Deposition and Characterization

**Figure 1:**
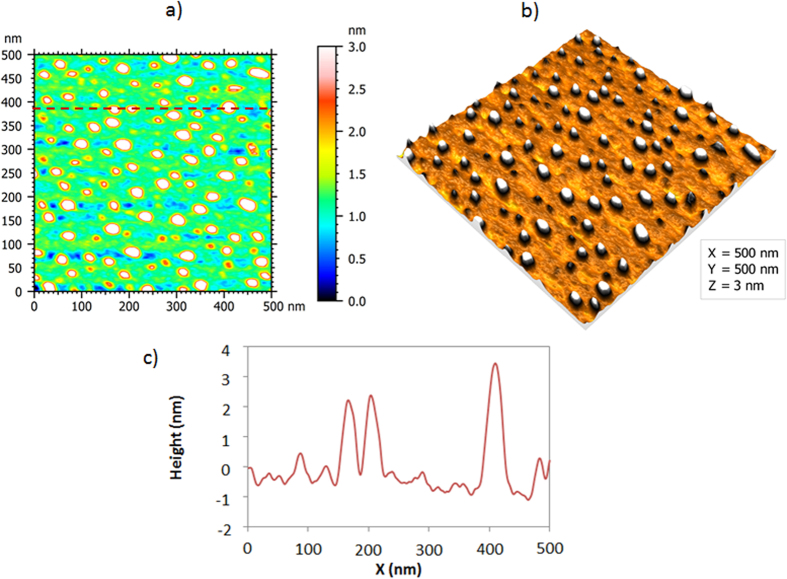
ZnO nanoislands topography after performing AFM tip de-convolution. (**a**) 2D AFM image of nanoislands. (**b**) 3D AFM image of the nanoislands. (**c**) Cross-sectional profile of the islands across the dashed line shown in (**a**).

**Figure 2:**
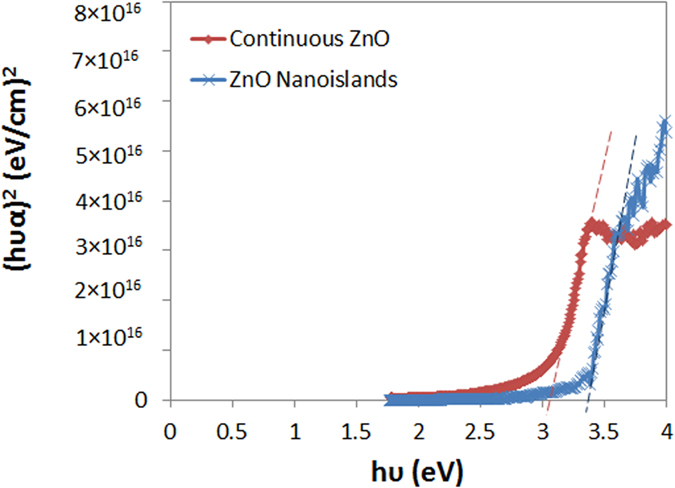
Kubelka-Munk plot of the ZnO nanoislands and continuous layer^69^.

The ZnO islands are deposited by 20 ALD cycles at 180 °C on a lightly doped p-type Si substrate. The 2D and 3D Atomic Force Microscopy (AFM) topography plots (0.5 × 0.5 μm) of the ZnO islands after performing tip de-convolution^25^ are depicted in Fig. 1a,b. Moreover, Fig. 1c shows the cross-sectional profile along the dashed line in Fig. 1a. The AFM images show that the nanoislands are dispersed and separated from each other and have a width range from 8.5–30 nm (average ~20 nm) and an average thickness of ~3 nm. The transmittance and reflectance spectra are measured and the Kubelka-Munk function is used to extract the bandgap of the islands as shown in Fig. 2^26^. The function consists of plotting (hυα)^2^ vs. hυ where hυ is the photon energy and α is the absorption coefficient^27^,^28^,^29^,^30^,^31^. In fact,

In the parabolic band structure, the absorption coefficient α and energy gap E_g_ of a direct-band gap material are related through the known following equation^32^:


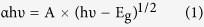


where hυ is the photon energy and A is a proportionality constant.

The absorption coefficient can be extracted from the transmittance and reflectance spectra using the following equation:


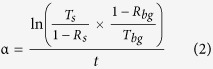


where t is the thickness of the semiconductor layer, T_s_ and R_s_ are the transmittance and reflectance measured using the UV-Vis-NIR spectrophotometer on the sample, and T_bg_ and R_bg_ are the transmittance and reflectance of the background (without the semiconductor layer).

Therefore, by measuring the needed reflectance and transmittance spectra, the absorption coefficient can be calculated and the bandgap can be therefore easily extracted using equation 1.

The same measurements are conducted on an 18-nm continuous ZnO layer. Figure 2 shows that the extracted bandgap of the continuous layer is 3.1 eV while this value for the islands increases to 3.35 eV due to quantum confinement of charges in 1D. In fact, a thick-layer of semiconductor usually shows bulk properties of the material. As the dimension of the layer is reduced to be comparable to the Bohr radius of the excitons of the material, quantum confinement effects take place. If only one dimension of the material is reduced (such as thickness resulting in a thin layer), then quantum confinement effects in 1 dimension are expected. This means that the material is a 2D nanostructure (quantum well): the electrons/holes have 2 degrees of freedom in x and y, however, they are confined in the z-direction into specific discrete energy levels which depend on the size of the quantum well (i.e. thickness of the layer). The thickness of the islands is around 3-nm which is close to the Bohr radius of the excitons in ZnO (~2.3 nm) but the width is much larger (~20 nm). Therefore, quantum confinement of electrons along the thickness of the islands is expected.

**Figure 3:**
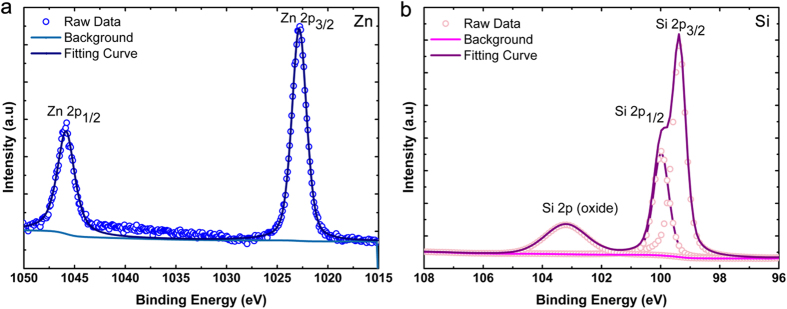
Core level spectra of (**a**) Zn 2p3/2 and (**b**) Si 2p3/2 recorded on Si-ZnO heterojunction. All peaks have been fitted to Voigt line shapes using a Shirley background.

**Figure 4:**
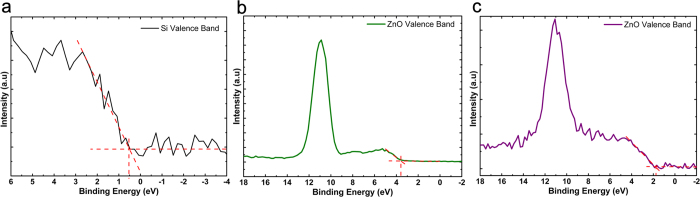
VB Spectra of (**a**) Si2p3/2 and Zn2p3/2 for (**b**) thick and (**c**) thin layers (VBM values are determined by extrapolating of leading edge to the base line).

However, Srikant *el al*. showed that the reported apparent bandgaps for ZnO of 3.1 eV and 3.2 eV in the literature are due to the existence of a valence band-donor level transition at ~3.15 eV which can dominate the absorption spectrum^33^. This value is close to the apparent bandgap reported in this work (3.1 eV) for the continuous 18-nm ZnO layer. In order to confirm the presence of defects levels in the deposited ZnO, X-ray photoelectron spectroscopy (XPS) measurements are conducted on the continuous and islands ZnO samples. First the energy difference between Zn2p and Si2p core levels (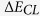) in the Si-ZnO (heterojunction) sample is measured and found to be 923.46 eV as shown in Fig. 3a,b. On the other hand, 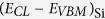, the energy difference of the core level to valance band maximum (VBM) for Si is determined as 98.85 eV from Fig. 4a. Whereas, this amount is found to be 1021.15 eV and 1019.23 eV for 3 nm and 18 nm thick ZnO layer, respectively. It should be noted that, as it can be implied from Fig. 4b,c, the value of *E*_*VBM*_ shows a considerable change from 3.6 eV to 1.68 eV when we move from continuous layer to nanoisland morphology. This is also in line with our assumption about emergence of quantum confinement effects for ultrathin ZnO layer. All obtained data are summarized in Table 1. Finally, the valence and conduction band offsets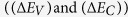 of Si/ZnO junction are calculated and summarized in Table 2. The results shown in Table 2 show that, due to quantum confinement, the electron affinity of the ZnO islands is reduced.

**Figure 5:**
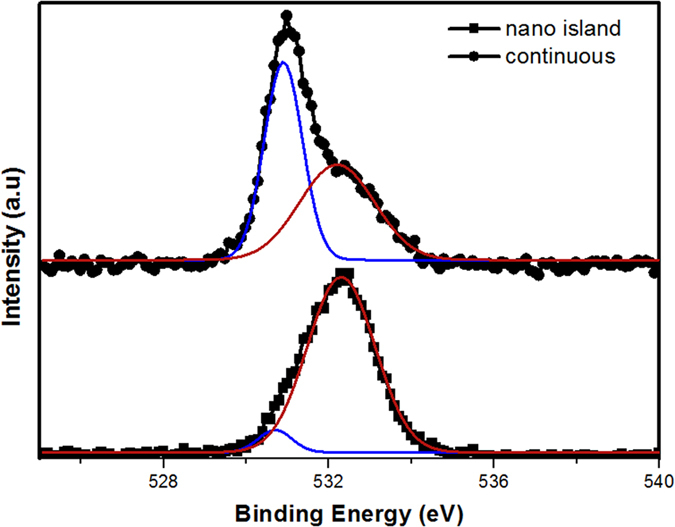
O 1 s spectra of ZnO layer for nano island and continuous films. Blue cure represents L_O_ signal and red one shows V_O_ peak.

To explain this drastic shift in *E*_*VBM*_ of nanoisland case, we need to analyze core-level spectrum of O 1 s for ZnO layer. For this aim, this spectrum is deconvoluted to two main peaks located at ~530.7 eV corresponding to lattice oxygen (L_O_) and ~532.3 eV due to chemisorbed oxygen species at the lattice defect sites and oxygen vacancies (V_O_)^34^. The existence of oxygen species such as –OH, –CO, adsorbed H_2_O and/or O_2_ on the surface generally produces a peak around 532.3 eV^25^,^27^. Looking at Fig. 5, we can see that the area under chemisorbed oxygen peak is much higher for nanoisland case compared to continuous layer. Due to the fact that defect sites and grain boundaries are subjected to the chemisorption of the above ions, this confirms that the nanoisland ZnO layer contains much more oxygen defects compared to the continuous one. These oxygen-contained molecules are chemisorbed on the ZnO surface by capturing free electrons of the host material^34^,^35^,^36^. Therefore, in the vicinity of the surface, these oxygen radicals decline the density of free carriers and deplete the surface electron states. This leads to formation of the space charge region which in turn leads to band bending near the surface region. Based on previous studies, Si (p-type)/ZnO (n-type) heterostructure have a surface band bending which is downward in Si side and upward in ZnO. Therefore, both quantum confinement effect and surface band bending cause this drastic change in the *E*_*VBM*_ level of the nanoisland structure.

**Figure 6:**
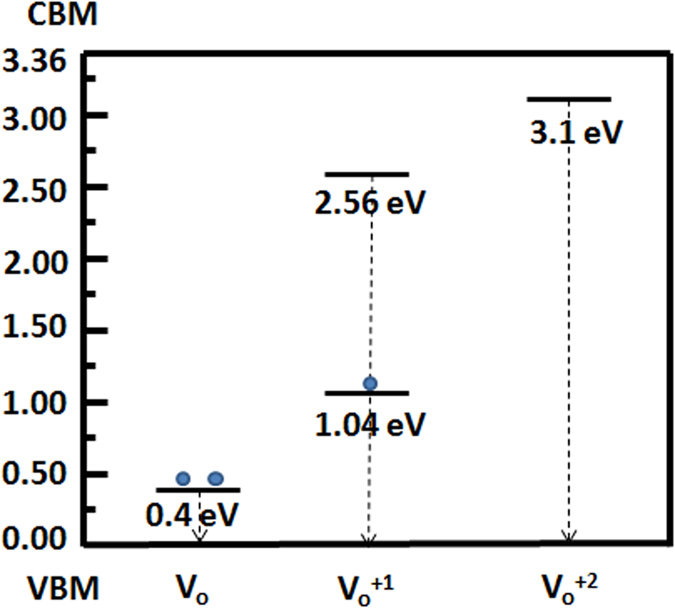
Oxygen Vacancy defects in the bulk ZnO.

In principle there are four main types of defects on ZnO which can be classified as: oxygen vacancies (V_O_’s), zinc vacancies (V_Zn_’s) (surface defects), interstitials (Zn_i_ and O_i_), and antisites which are mostly formed in the bulk of material. The fact that which of these defects is dominant depends on the preparation method of the material under Zn reach conditions VO’s are the most favorable defects to form. This conclusion has been already confirmed by XPS measurement in which the O1s spectra show the existence of V_O_’s on the as prepared samples, while there is no sign related to Zn_i_ in Zn2p spectra. These oxygen vacancies are of three types: Vo (neutral, filled with 2 electrons, usually found at 0.4 eV from the valence band) V_O_^+1^ (charged, filled with one electron or unoccupied and doubly degenerate, usually found at 1.04 and 2.56 eV from the valence band, respectively) and V_O_^+2^ (charged, unoccupied and doubly degenerate, usually found at around 3.1–3.2 eV from the valence band)^34^,^36^,^37^. Figure 6 shows these estimated defect levels within the bandgap of our bulk ZnO^37^,^38^. For the nanoislands, the bandgap is expected to be increased due to quantum confinement. The Kubelka-Munk plot showed a valence band-oxygen vacancy transition at around 3.35 eV since the bandgap of 2D ZnO nanostructures is reported to be around 3.62 eV^39^. Moreover, the XPS results show that the density of defects in the nanoislands is much larger than in the bulk ZnO case. In addition, in order to estimate the bandgap of the ZnO nanoislands, a back-of-the-envelope method for 2D nanostructures is used by applying the following equation^40^:


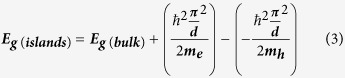


where E_g(islands)_ and E_g(bulk)_ are the bandgaps of the ZnO islands and bulk ZnO, respectively, m_h_ and m_e_ are the effective masses of holes and electrons in ZnO respectively, (0.59m_0_ and 0.24m_0_, respectively)^41^, d is the thickness of the islands (3-nm) and ħ is the reduced Plank’s constant. The calculated bandgap of the islands is 3.604 eV which is consistent with the reported bandgap of 2D- ZnO nanostructures (3.62 eV) in the literature^39^.

**Figure 7:**
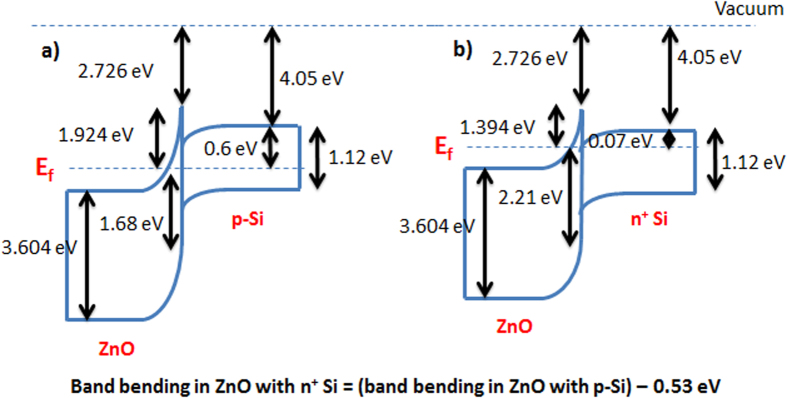
Energy band diagram of the ZnO islands/Si heterojunction. (**a**) With p-type Si. (**b**) With highly doped n-type Si.

Based on the XPS measurements, the band diagrams of the ZnO islands/Si heterojunctions with p-type Si and highly doped n-type Si are shown in Fig. 7a,b, respectively. The figures show that the electron affinity of the ZnO is reduced to 2.726 eV which is an expected effect of quantum confinement. In addition, the band bending at the interface to align the Fermi level creates a barrier for trapped holes in the ZnO which helps in reducing the charge leakage probability as will be explained with more details in the following.

### Memory Devices characterization

**Figure 8:**
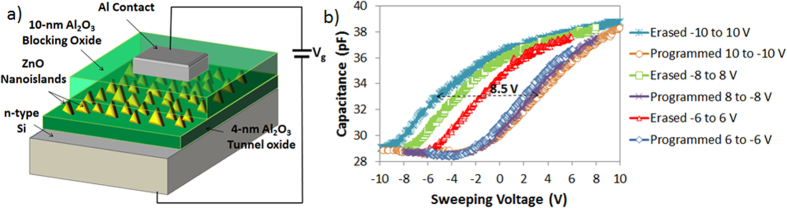
Memory device with ZnO nanoislands. (**a**) Cross-sectional illustration of the memory. (**b**) High-frequency (1 MHz) C-V measurements of erased and programmed states of the memory devices.

**Figure 9:**
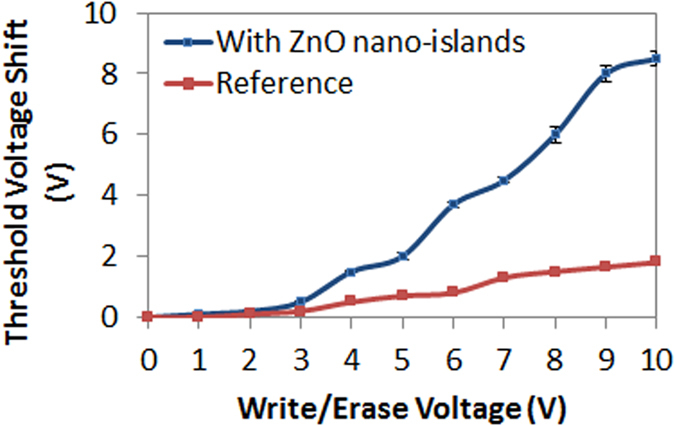
Threshold voltage shift vs. program/erase voltage for the memory with nanoislands and reference memory.

The memory devices are fabricated on highly doped n^+^ type Si wafer (111) (Antimony doped, 15–20 mΩ-cm). Atomic layer deposited Al_2_O_3_ is used for both tunnel and blocking oxides. ZnO nanoislands are used as the charge trapping layer. The 250-nm Al e-beam deposited contacts are patterned using a shadow mask. The deposition of the active layer of the memory in a single ALD step greatly reduces the contamination probability^42^. Figure 8a shows the cross-section illustration of the fabricated memory devices. A reference memory without ZnO nanoislands is also fabricated. The devices are electrically characterized by measuring the high-frequency (1 MHz) C-V_gate_ characteristics. A gate voltage of 6 V is first applied for 1 s and the memory C-V is measured, followed by −6 V for 1 s. A large memory window of 4 V is obtained as shown in Fig. 8b. Similar C-V measurements are conducted at different program/erase voltages. It is observed that the memory devices are being programmed by applying a negative gate voltage. Moreover, a very large threshold voltage (V_t_) shift of 8.5 V is obtained at −10/10 V program/erase voltage which proves that the ultra-thin ZnO islands have a large density of charge trapping states while a very negligible shift is obtained in the reference cells as shown in Fig. 9.

**Figure 10:**
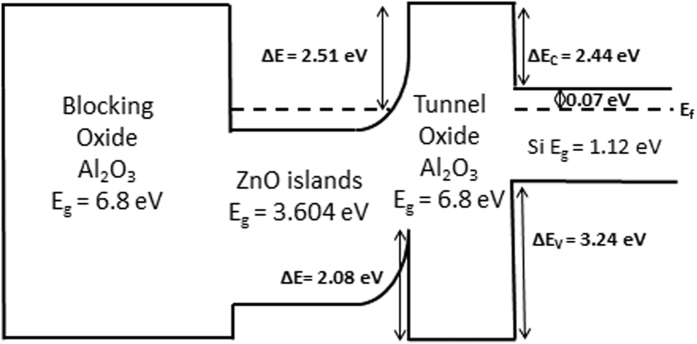
Energy band diagram of the memory with ZnO nanoislands showing the Fermi level.

The energy band diagram of the memory cells are constructed as shown in Fig. 10 based on the XPS measurements and reported values of the band offsets and bandgaps of the different materials^43^,^44^,^45^. Therefore, during the program operation when a negative gate voltage is applied, the electrons in V_O_ and V_O_^+^ states gain enough energy and get emitted to the channel while holes in the channel gain enough energy and tunnel through the tunnel oxide and get trapped within the deep quantum well formed by the valence band offsets. This will cause the programmed CV characteristic of the memory to shift to the left. During the erase operation, the electrons from the channel tunnel back into the trap states (oxygen vacancies + quantum well) within the ZnO bandgap while holes in the quantum well tunnel back into the channel which will cause the CV characteristic of the memory to shift back to the right.

**Figure 11:**
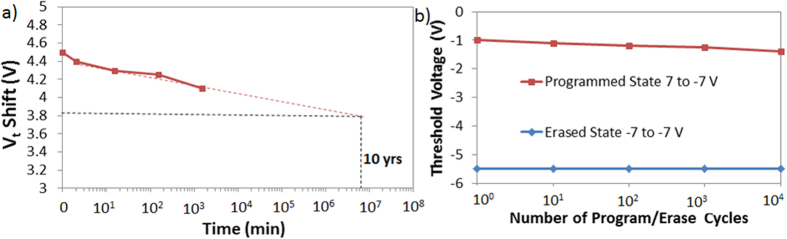
Memory characteristics (**a**) Retention characteristic of the memory initially programmed/erased at −7/7 V. (**b**) Endurance characteristic up to 10^4^ cycles.

In addition, the retention characteristics of the memory cells is studied by first programming/erasing the memory at −7/7 V and reading the memory state in time as shown in Fig. 11a. The retention characteristic extrapolated to 10 years shows a 3.85 V V_t_ shift where 14.4% of the initial charge is lost in 10 years. To explain the good retention characteristic, we need to analyze the tunneling probability of the electrons from the channel back into the oxygen vacancies and the tunneling of the holes from the ZnO quantum well back into the channel with no applied gate voltage. First of all, for the electrons available in the channel to tunnel into the oxygen vacancies and quantum well, they must overcome a large barrier ΔE_C_ of 2.44 eV (as shown in Fig. 10), therefore the tunneling/emission probability is very low especially when no electric field is applied (retention operation). Second, for the holes stored in the ZnO quantum well to leak back, they need to overcome a barrier ΔE of 2.08 eV caused by the reduction of the electron affinity of ZnO (as shown in the energy band diagram Fig. 10) which is also too large for holes which have a larger effective tunneling mass than electrons. Moreover, the endurance characteristic of the memory is analyzed by programming and erasing the cells at −7/7 V up to 10^4^ times while observing the change in the V_t_ shift. As shown in Fig. 11b, after 10^4^ cycles, a large memory window of 4 V is still measured which means that ~11% of the initial charge is lost. This outstanding endurance characteristic shows that such memory structure is promising for future memory devices.

## Discussion

The growth per cycle in ALD which is the amount of material deposited in an ALD reaction cycle, is a function of three parameters: the reactants, the reaction temperature, and the surface where the reactions occur^46^. A constant growth per cycle has been a paradigm in ALD through decades^47^,^48^. Recently it has been realized that while depositing new material, the ALD process changes the characteristics of the substrate, therefore the growth per cycle does not need to be constant^49^,^50^,^51^,^52^. Moreover, it has been shown that in the first ~20 ALD cycles (depending on the material), 3D islands form in the so called Volmer-Weber growth mode^53^. In this growth mode, it has been observed a generic stress evolution from compressive to tensile, then back to compressive stress as the film thickened. The tensile stress is attributed to the impingement and coalescence of growing islands: islands strain to close the gap between them and replace the free surfaces with a low energy grain boundary^54^,^55^. However, if the growth process is interrupted at the islands stage, then the tensile stress will be relaxed and the mechanism depends sensitively on the process conditions, on whether the film is continuous or discontinuous, and on the film/substrate interfacial strength^56^. Some of the reported relaxation mechanisms include interfacial shear, selective relaxation consistent with surface-diffusion-based mechanisms, and dislocations to name a few^57^,^58^,^59^,^60^,^61^. In our case, since the memory showed a much larger memory window and therefore a larger charge trapping states density than in the case of continuous ZnO layer with almost the same thickness as reported in our previous work where a negligible V_t_ shift was obtained at 10/−10 V program erase voltage^19^, then the tensile stress in the nanoislands is expected to be relaxed through a mechanism that adds trap states in ZnO such as dislocations. In addition, oxygen vacancies are reported to relax the stress in ZnO^62^,^63^. Therefore, the observed larger density of oxygen vacancies in the nanoislands than in the continuous ZnO could be due to the stress relaxation mechanism in the islands. As a matter of fact, the introduction of large densities of defects into semiconductors is needed in many applications. For example, the introduction of deep level defects moves the Fermi Level close to midgap and greatly increases the resistivity is desirable for photoconductors since it reduces the dark current. Another feature of high defect densities is an enhanced optical absorption in the spectral region below the edge for direct transitions due to the introduction of new states and the relaxation of selection rules for indirect transitions. A third advantage of the use of high defect density materials is the ease of fabrication of ohmic contacts. And for memory devices, the introduction of defects introduces additional trap states which enlarge the memory window.

Moreover, when programming the memory at higher negative voltages, the C-V curves shifts to the left indicating that holes are being stored and electrons are being removed. While at higher positive gate voltages, the C-V curves shift to the right indicating that electrons are being stored and holes are being removed. The reduction of the electron affinity of the ZnO due to quantum confinement increases the valence band offset between the charge trapping layer and surrounding tunnel and blocking oxides, and therefore, additional hole trap states are available. Additionally, the oxygen vacancies states contribute in trapping electrons. The charge trap states density can be calculated using the equation ΔN = ΔV_t_ × C_acc_, where ΔV_t_ is the threshold voltage shift and C_acc_ is the accumulation capacitance per unit area. ΔN is found to be 3.1 × 10^13^ cm^−2^.

**Figure 12:**
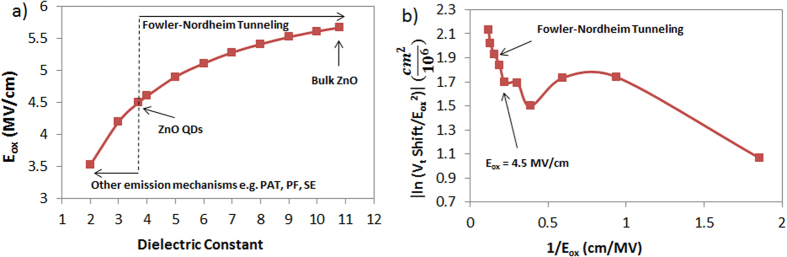
(**a**) Electric field across the tunnel oxide vs. the dielectric constant of the ZnO with an applied gate V_g_ = 6 V. (**b**) Natural logarithm of the threshold voltage shift over the squared electric field across the tunnel oxide vs. the reciprocal of the electric field. Linear trends shows that F-N tunneling is dominant at E_ox_ > 4.5 MV/cm.

In order to study the charge emission mechanism during the program/erase operations, the electric field across the tunnel oxide needs to be calculated using Gauss’s law. However, due to quantum confinement, the dielectric constant of the ZnO is expected to be lowered. Moreover, it is known that Fowler-Nordheim is the dominant tunneling mechanism across Al_2_O_3_ when the electric field is >~4.5 MV/cm^64^,^65^,^66^. Therefore, the electric field across the tunnel oxide is calculated for different ZnO dielectric constant values. It is worth noting that the dielectric constant of bulk ZnO is around 10.8 while ZnO with quantum confinement effects in 3D (ZnO quantum dots) has a reported dielectric constant of around 3.7^67^. Thus, the dielectric constant of the 2D ZnO islands is expected to be in this range. Figure 12a shows the calculated electric field across the tunnel oxide when V_g_ = 6 V vs. the ZnO dielectric constant, the figure shows that for dielectric constants above ~3.7, E_ox_ > 4.5 MV/cm, therefore, Fowler-Nordheim is expected to be the dominant charge emission mechanism in the memory device when the applied gate voltage is equal or greater than 6 V. In fact, F-N tunneling is the tunneling mechanism which requires the highest electric field across the tunnel oxide in order to create a triangular barrier through which charges can tunnel. For dielectric constants below 3.7, E_ox_ < 4.5 MV/cm, and other emission mechanisms would be dominant such as Phonon-Assisted Tunneling (PAT), Poole-Frenkel (PF), and Schottky emission (SE)^68^. Moreover, for a dielectric constant of 3.7, the logarithm of the threshold voltage shift over the squared electric field across the tunnel oxide vs. the reciprocal of the electric field is plotted in Fig. 12b and the linear trend at E_ox_ > 4.5 MV/cm which corresponds to V_g_ = 6 V confirms that Fowler-Nordheim tunneling is the dominant emission mechanism.

In summary, 3-nm-thick ZnO nanoislands are deposited by ALD and characterized. The results show that due to quantum confinement in 1D, the bandgap of the nanoislands increases and the electron affinity is reduced. Moreover, memory devices with ZnO nanoislands charge trapping layer fabricated by a single ALD step are demonstrated. The measurements show that a large memory window of 4 V can be obtained at low operating voltages due to holes storage and electrons removal from the nanoislands. The increased charge trapping states density in the islands is due to additional defect states (oxygen vacancies) due to the tensile stress relaxation in the islands. Moreover, the reduction of the electron affinity of the ZnO created a deep barrier for the trapped holes in ZnO, improving the retention characteristic of the memory. The excellent retention and endurance characteristics of the memory indicate that ZnO nanoislands are promising in future nonvolatile charge trapping memory devices.

## Methods

### Materials

The following materials are used as precursors in the ALD system: Diethyl zinc (DEZ) and Trimethylaluminium (TMA - Al(CH_3_)).

### Fabrication and characterization of the nanoislands

The nanoislands are deposited on highly doped n-type Si (111) which was first cleaned using HF acid to remove the native oxide. The same deposition is performed on glass wafers in order to measure the transmittance and reflectance spectra of the islands. The transmittance and reflectance spectra are obtained using a LAMBDA 1050 UV-Vis-NIR spectrophotometer. The bandgap of the islands is obtained by first plotting (hυα)^2^ vs. hυ (since ZnO is a direct bandgap material) where hυ is the photon energy and α is the absorption coefficient. The bandgap is found by plotting the intersection between the x-axis and the step edge as shown in Fig. 2.

### Fabrication and characterization of the memory devices

On highly doped n-type Si (111), 4-nm Al_2_O_3_ tunnel oxide is deposited by ALD using an Oxford Flexal system at 300 °C, followed by 20 cycles of ZnO at 180 °C. The deposition of the ZnO nanoislands consists of the following cycles: DEZ dose time: 20 ms, Ar purge: 10 s, H_2_O dose: 100 ms, Ar purge: 10 s. Then, 10-nm Al_2_O_3_ blocking oxide is deposited at 300 °C and 80 mtorr. The contacts are deposited using a Temescal e-beam deposition system at 3 × 10^−6^ torr and patterned using a shadow mask. The electrical characterization of the devices is conducted using an Agilent B1505A semiconductor device analyzer connected to a Signatone probe station.

### AFM Imaging

For imaging, AC-in-Air imaging mode was used. Si based AFM cantilevers with Al reflective coating. The tips radii are ~9 nm according to the manufacturer specifications.

## Additional Information

**How to cite this article:** El-Atab, N. *et al*. ~3-nm ZnO Nanoislands Deposition and Application in Charge Trapping Memory Grown by Single ALD Step. *Sci. Rep.* **6**, 38712; doi: 10.1038/srep38712 (2016).

**Publisher's note:** Springer Nature remains neutral with regard to jurisdictional claims in published maps and institutional affiliations.

## Figures and Tables

**Table 1:** Binding energies obtained by X-ray photoelectron spectroscopy measurements.

Sample	Region	Binding Energy (eV)
Si	VBM	0.52
ZnO (3 nm)	VBM	1.68
ZnO (18 nm)	VBM	3.6
Si/ZnO	CL Si 2p^3/2^	99.37
	CL Zn 2p^3/2^	1022.83

**Table 2:** Conduction and valence band offsets between continuous and ZnO nanoislands on Si obtained by X-ray photoelectron spectroscopy measurements.

	Continuous ZnO (18-nm)	ZnO islands (3-nm)
E_g_	3.36 eV	3.604 eV
E_C (Si)_ - E_C (ZnO)_	0.84 eV	−1.324 eV
E_V (Si)_ - E_V (ZnO)_	3.08 eV	1.16 eV

## References

[b1] Hu Q., Wang J., Zhao Y. & Li D. A light-trapping structure based on Bi_2_O_3_ nano-islands with highly crystallized sputtered silicon for thin-film solar cells. Optics express. 19(101), A20–A27 (2011). 21263708 10.1364/OE.19.000A20

[b2] Hu Q., Wang J., Cao Y., Zhao Y. & Li D. Light-trapping enhancement based on Ga_2_O_3_ nano-islands coated glass substrate. Solar Energy 86(3), 855–859 (2012).

[b3] Qi J., Olmedo M., Zheng J. G. & Liu J. Multimode resistive switching in single ZnO nanoisland system. Scientific reports 3 (2013). 10.1038/srep02405 PMC3740279 23934276

[b4] Zaretski A. V. . Metallic Nanoislands on Graphene as Highly Sensitive Transducers of Mechanical, Biological, and Optical Signals. Nano letters 16(2), 1375–1380 (2016). 26765039 10.1021/acs.nanolett.5b04821 PMC4751512

[b5] Sun X. & Li H. Gold nanoisland arrays by repeated deposition and post-deposition annealing for surface-enhanced Raman spectroscopy. Nanotechnology 24(35), 355706 (2013). 23942082 10.1088/0957-4484/24/35/355706

[b6] Alkhatib A. & Nayfeh A. A Complete Physical Germanium-on-Silicon Quantum Dot Self-Assembly Process. Scientific reports 3 (2013). 10.1038/srep02099 PMC3695558 23807261

[b7] Bacaksiz E. . The effect of zinc nitrate, zinc acetate and zinc chloride precursors on investigation of structural and optical properties of ZnO thin films. J. Alloy. Compd. 466, 447‒450 (2008).

[b8] Wang J. . Synthesis and characterization of multipod, flower-like, and shuttle-like ZnO frameworks in ionic liquids. Mater. Lett. 59, 1405‒1408 (2005).

[b9] Özgür Ü. . A comprehensive review of ZnO materials and devices. Journal of applied physics 98(4), 041301 (2005).

[b10] El-Atab Nazek, . Diode behavior in ultra-thin low temperature ALD grown zinc-oxide on silicon. AIP Advances 3(10), 102119 (2013).

[b11] Ulusoy T. G., Ghobadi A. & Okyay A. K. Surface Engineered Angstrom Thick ZnO-sheathed TiO_2_ Nanowires as Photoanode for Performance Enhanced Dye-sensitized Solar Cells. Journal of Materials Chemistry A 2, 16867–16876, doi: 10.1039/c4ta03445g (2014).

[b12] Ghobadi A. . Enhanced Performance of Nanowire-Based All-TiO_2_ Solar Cells using Subnanometer-Thick Atomic Layer Deposited ZnO Embedded Layer. Electrochimica Acta 157, 23, doi: 10.1016/j.electacta.2015.01.079 (2015).

[b13] Battal E. . Atomic Layer Deposited Zinc-Oxide as Tunable Uncooled Infrared Microbolometer Material. Physica Status Solidi A, doi: 10.1002/pssa.201431195 (2014).

[b14] Oruc F. B. . Low Temperature Atomic Layer Deposited ZnO Photo Thin Film Transistors. Journal of Vacuum Science and Technology A 33, 01A105, doi: 10.1116/1.4892939 (2015).

[b15] Leskelä M. & Ritala M. Atomic layer deposition (ALD): from precursors to thin film structures. Thin solid films. 409(1), 138–146 (2002).

[b16] Choy K. L. Chemical vapour deposition of coatings. Progress in materials science. 48(2), 57–170 (2003).

[b17] Wu M. K., Chen M. J., Tsai F. Y., Yang J. R. & Shiojiri M. Fabrication of ZnO nanopillars by atomic layer deposition. Materials transactions. 51(2), 253–255 (2010).

[b18] Nayfeh A., Okyay A. K., El-Atab N., Cimen F. & Alkis S. Transparent Graphene Nanoplatelets for Charge Storage in Memory Devices. Invited 226th ECS meeting 2014. no. 37, p. 1879–1879 (2014).

[b19] El-Atab N. . Enhanced memory effect via quantum confinement in 16 nm InN nanoparticles embedded in ZnO charge trapping layer. Applied Physics Letters 104(25), 253106 (2014).

[b20] El-Atab N. & Nayfeh A. 1D vs. 3D quantum confinement in 1–5 nm ZnO nanoparticles agglomerations for application in charge trapping memory devices. Nanotechnology 27, 275205 (2016). 27232717 10.1088/0957-4484/27/27/275205

[b21] Nayfeh A., Okyay A. K., El-Atab N., Ozcan A. & Alkis S. Low Power Zinc-Oxide Based Charge Trapping Memory with Embedded Silicon Nanoparticles. Invited, 226th ECS meeting 2014 no. 46, p. 2143–2143 (2014).

[b22] El-Atab N. . Memory effect by charging of ultra-small 2-nm laser-synthesized solution processable Si-nanoparticles embedded in Si-Al2O3-SiO2 structure. Phys. Status Solidi A 212(8), 1751–1755 doi: 10.1002/pssa.201431802 (2015).

[b23] El-Atab N., Turgut B. B., Okyay A. K., Nayfeh M. & Nayfeh A. Enhanced non-volatile memory characteristics with Quattro-layer graphene-nanoplatelets vs. 2.85-nm Si-nanoparticles with asymmetric Al_2_O_3_/HfO_2_ tunnel oxide. Nanoscale Res. Lett. 10(1), 248, doi: 10.1186/s11671-015-0957-5 (2015). PMC4456595 26055483

[b24] Oruç F. B., Cimen F., Rizk A., Ghaffari M., Nayfeh A. & Okyay A. K. Thin-film ZnO charge-trapping memory cell grown in a single ALD step. IEEE Electron Device Letters 33(12), 1714–1716 (2012).

[b25] MountainsMap^®^ software by Digital Surf.

[b26] El-Atab N. . Growth of ~3-nm ZnO Nano-islands Using Atomic Layer Deposition. To be published and presented at *2016 IEEE Nanotechnology Conference* Sendai, Japan, August 22–25 (2016).

[b27] Liao L. . Efficient solar water-splitting using a nanocrystalline CoO photocatalyst. Nature nanotechnology 9(1), 69–73 (2014). 10.1038/nnano.2013.272 24336404

[b28] Kim H. S. . Lead iodide perovskite sensitized all-solid-state submicron thin film mesoscopic solar cell with efficiency exceeding 9%. Scientific reports 2 (2012). 10.1038/srep00591 PMC3423636 22912919

[b29] Lee J. S., You K. H. & Park C. B. Highly photoactive, low bandgap TiO_2_ nanoparticles wrapped by graphene. Advanced Materials 24(8), 1084–1088 (2012). 22271246 10.1002/adma.201104110

[b30] Gao F. . Visible‐Light Photocatalytic Properties of Weak Magnetic BiFeO_3_ Nanoparticles. Advanced Materials 19(19), 2889–2892 (2007).

[b31] Goodall J. B. . Optical and photocatalytic behaviours of nanoparticles in the Ti–Zn–O binary system. RSC Advances 4(60), 31799–31809 (2014).

[b32] Smith R. A. Semiconductors. Cambridge University Press: Cambridge, 2^nd^ ed. (1978).

[b33] Srikant V. & Clarke D. R. On the optical band gap of zinc oxide. Journal of Applied Physics 83(10), 5447–5451 (1998).

[b34] Kayaci F. . Selective isolation of the electron or hole in photocatalysis: ZnO–TiO_2_ and TiO_2_–ZnO core–shell structured heterojunction nanofibers via electrospinning and atomic layer deposition. Nanoscale. 6(11), 5735–5745 (2014). 24664354 10.1039/c3nr06665g

[b35] Rogé V. . Improvement of the photocatalytic degradation property of atomic layer deposited ZnO thin films: the interplay between film properties and functional performances. Journal of Materials Chemistry A. 3(21), 11453–11461 (2015).

[b36] Kayaci F., Vempati S., Donmez I., Biyikli N. & Uyar T. Role of zinc interstitials and oxygen vacancies of ZnO in photocatalysis: a bottom-up approach to control defect density. Nanoscale. 6(17), 10224–10234 (2014). 25056654 10.1039/c4nr01887g

[b37] Ulusoy T. G., Ghobadi A. & Okyay A. K. Surface engineered angstrom thick ZnO-sheathed TiO_2_ nanowires as photoanodes for performance enhanced dye-sensitized solar cells. Journal of Materials Chemistry A. 2(40), 16867–16876 (2014).

[b38] Hu J. & Pan B. C. Electronic structures of defects in ZnO: hybrid density functional studies. The Journal of chemical physics 129(15), 154706 (2008). 19045217 10.1063/1.2993166

[b39] Samanta P. K. Characteristics of benzene assisted grown ZnO nanosheets. Science of Advanced Materials. 4(2), 219–226 (2012).

[b40] Klimov V. I. (Ed.). Semiconductor and metal nanocrystals: synthesis and electronic and optical properties. CRC Press (2003).

[b41] Jagadish C. & Pearton S. J. (Eds). Zinc oxide bulk, thin films and nanostructures: processing, properties, and applications. Elsevier (2011).

[b42] Oruc F. B. . Thin Film ZnO Charge Trapping Memory Cell Grown in a Single ALD Step. IEEE Electon Device Letters 33, 1714–1716, doi: 10.1109/LED.2012.2219493 (2012).

[b43] Wilk G. D., Wallace R. M. & Anthony J. M. High-κ gate dielectrics: Current status and materials properties considerations. Journal of applied physics 89(10), 5243–5275 (2001).

[b44] Huang M. L. . Energy-band parameters of atomic-layer-deposition Al_2_O_3_/InGaAs heterostructure. Applied physics letters 89(1), 012903 (2006).

[b45] Lu H. L. . Band alignment and interfacial structure of ZnO/Si heterojunction with Al_2_O_3_ and HfO_2_ as interlayers. Applied Physics Letters. 104(16), 161602 (2014).

[b46] Goodman C. H. & Pessa M. V. Atomic layer epitaxy. Journal of applied physics. 60(3), R65–R82 (1986).

[b47] Leskelä M. & Ritala M. Atomic layer deposition chemistry: recent developments and future challenges. Angewandte Chemie International Edition. 42(45), 5548–5554 (2003). 14639717 10.1002/anie.200301652

[b48] Suntola T. Atomic layer epitaxy. Materials Science Reports. 4(5), 261–312 (1989).

[b49] Puurunen R. L. Growth per cycle in atomic layer deposition: a theoretical model. Chemical Vapor Deposition. 9(5), 249–257 (2003).

[b50] Satta A. . Growth mechanism and continuity of atomic layer deposited TiN films on thermal SiO_2_. Journal of applied physics. 92(12), 7641–7646 (2002).

[b51] Puurunen R. L. Analysis of hydroxyl group controlled atomic layer deposition of hafnium dioxide from hafnium tetrachloride and water. Journal of applied physics 95(9), 4777–4786 (2004).

[b52] Alam M. A. & Green M. L. Mathematical description of atomic layer deposition and its application to the nucleation and growth of HfO_2_ gate dielectric layers. Journal of applied physics. 94(5), 3403–3413 (2003).

[b53] Puurunen R. L. & Vandervorst W. Island growth as a growth mode in atomic layer deposition: A phenomenological model. Journal of Applied Physics. 96(12), 7686–7695 (2004).

[b54] Nix W. D. & Clemens B. M. Crystallite coalescence: A mechanism for intrinsic tensile stresses in thin films. Journal of Materials Research 14(08), 3467–3473 (1999).

[b55] Freund L. B. & Chason E. Model for stress generated upon contact of neighboring islands on the surface of a substrate. Journal of Applied Physics. 89(9), 4866–4873 (2001).

[b56] Floro J. A. . The dynamic competition between stress generation and relaxation mechanisms during coalescence of Volmer–Weber thin films. Journal of Applied Physics. 89(9), 4886–4897 (2001).

[b57] Floro J. A. . Novel SiGe island coarsening kinetics: Ostwald ripening and elastic interactions. Physical review letters. 84(4), 701 (2000). 11017351 10.1103/PhysRevLett.84.701

[b58] Family F. & Meakin P. Scaling of the droplet-size distribution in vapor-deposited thin films. Physical review letters. 61(4), 428 (1988). 10039330 10.1103/PhysRevLett.61.428

[b59] Thouless M. D. Effect of surface diffusion on the creep of thin films and sintered arrays of particles. Acta metallurgica et materialia. 41(4), 1057–1064 (1993).

[b60] Floro J. A. . The dynamic competition between stress generation and relaxation mechanisms during coalescence of Volmer–Weber thin films. Journal of Applied Physics. 89(9), 4886–4897 (2001).

[b61] Kobrinsky M. J. & Thompson C. V. The thickness dependence of the flow stress of capped and uncapped polycrystalline Ag thin films. Applied physics letters. 73(17), 2429–2431 (1998).

[b62] Janotti A. & Van de Walle C. G. Native point defects in ZnO. Physical Review B. 76(16), 165202 (2007).

[b63] Conchon F. . X-ray diffraction analysis of thermally-induced stress relaxation in ZnO films deposited by magnetron sputtering on (100) Si substrates. Thin Solid Films. 518(18), 5237–5241 (2010).

[b64] Groner M. D., Elam J. W., Fabreguette F. H. & George S. M. Electrical characterization of thin Al_2_O_3_ films grown by atomic layer deposition on silicon and various metal substrates. Thin Solid Films. 413(1), 186–197 (2002).

[b65] Shen Y. D. . Excellent insulating behavior Al_2_O_3_ thin films grown by atomic layer deposition efficiently at room temperature. Optoelectronic and advanced materials 6(5), 618–622 (2012).

[b66] Mahajan A. M., Khairnar A. G. & Thibeault B. J. Electrical properties of MOS capacitors formed by PEALD grown Al_2_O_3_ on silicon. Semiconductors 48(4), 497–500 (2014).

[b67] Lin K. F., Cheng H. M., Hsu H. C., Lin L. J. & Hsieh W. F. Band gap variation of size-controlled ZnO quantum dots synthesized by sol–gel method. Chemical Physics Letters 409(4), 208–211 (2005).

[b68] Ramesh S., Dutta S., Shankar B. & Gopalan S. Identification of current transport mechanism in Al_2_O_3_ thin films for memory applications. Applied Nanoscience 5(1), 115–123 (2015).

[b69] El-Atab, N. . Growth of ~3-nm ZnO Nano-islands Using Atomic Layer Deposition. *Proceedings of the 16^th^ IEEE International Conference on Nanotechnology (IEEE-Nano*), pp. 687–689 (2016).

